# Burden of invasive group a streptococcus infection in Australia: a systematic review and meta-analysis

**DOI:** 10.1007/s10096-026-05480-x

**Published:** 2026-04-10

**Authors:** Himali Ratnayake, Damon Eisen, Oyelola Adegboye, Anton Pak, Chanika Alahakoon, Mohabeer Teeluck, Gbeminiyi Richard Otolorin, Emma McBryde

**Affiliations:** 1 College of Medicine and Dentistry, James Cook University, Townsville, QLD Australia; 2 Menzies School of Health Research, Charles Darwin University, Northern Territory, Australia; 3 Public Health and Tropical Medicine, College of Medicine and Dentistry, James Cook University, Townsville, Queensland Australia; 4 Centre for Business and Economics of Health, The University of Queensland, Townsville, Queensland Australia; 5 College of Medicine and Dentistry, James Cook University, Cairns, Queensland Australia; 6 Australian Institute of Tropical Health and Medicine, James Cook University, Townsville, Queensland Australia; 7 Faculty of Health, Medicine, and Behavioural Sciences, University of Queensland, Brisbane, Australia; 8 Centre for Tropical Biosecurity, James Cook University, Townsville, Queensland Australia; 9 Burnet Institute, 85 Commercial Road, Melbourne, Victoria 3004, Australia

**Keywords:** Invasive Group A Streptococcus, Australia, Tropical, Infectious diseases, Streptococcus pyogenes

## Abstract

**Purpose:**

In this study, we aimed to estimate the burden of invasive Group A Streptococcus (iGAS) infection in Australia and describe the risk factors and outcomes across regions and populations.

**Methods:**

Studies published in six databases until October 2025 were included. The first study included in the systematic review was published in 1996. The review was registered in PROSPERO (CRD42023493112). The quality was assessed using The Joanna Briggs Institution checklist for prevalence studies. Common presentations, outcomes, risk factors, seasonality and emm types were systematically reviewed. A meta-analysis was conducted to estimate pooled incidence.

**Results:**

Forty-one articles were included in the systematic review and 10 were included in the meta-analysis. The pooled incidence of iGAS was 7.97 per 100,000 person-years (95% CI:4.21-15). Indigenous Australians experienced a higher incidence (26.31 per 100,000 person-years; 95% CI: 8.65–79.98) than non-Indigenous (4.58 per 100,000 person-years; 95% CI:2.67–7.85). Tropical regions also had a high incidence (29.22 per 100,000 person-years; 95% CI:18.04–47.34). Skin and Soft Tissue Infections (SSTIs) were the main primary focus of infection (35.6–71.3%). Around 6.6–16.5% of iGAS were Streptococcal Toxic Shock Syndrome (STSS). Mortality ranged from 5.6 to 13.8%. Old age, chronic diseases, IV drug use, Indigenous status and living in a tropical region were among the risk factors for iGAS while association with sex was mixed. The number of events reported increased in the third quarter, although not consistent. The predominant emm-types identified nationally were emm1, emm89, emm12, and emm4.

**Conclusion:**

iGAS poses a significant burden in Australia. Larger studies with less heterogeneity are needed to better understand the disease’s true impact and associated risk factors.

**Supplementary Information:**

The online version contains supplementary material available at 10.1007/s10096-026-05480-x.

## Introduction

Group A *Streptococci* (GAS), also known as *Streptococcus pyogenes*, causes a spectrum of diseases ranging from mild localised infections to severe conditions with high morbidity and mortality. GAS is recognised as a significant global health problem and is listed among the top 10 infectious causes of human mortality [1]. Skin or throat infections are mild and the commonest manifestations of GAS while sepsis, bacteraemia, bacteraemic pneumonia, necrotizing soft tissue infections (NSTI), Streptococcal Toxic Shock Syndrome (STSS), arthritis and severe skin and soft-tissue infections (SSTIs) are more invasive presentations [2, 3]. Immunologically mediated postinfectious sequelae, including acute post-streptococcal glomerulonephritis and rheumatic heart disease, are other important disease entities that may follow GAS infection, especially if they are untreated or inadequately treated.

Invasive GAS (iGAS) diseases have a high morbidity and mortality [4]. Every year, at least 663,000 new cases of iGAS are reported worldwide, with approximately 163,000 deaths [5]. The incidence of iGAS is increasing globally [6–9]. In December 2022, the World Health Organization (WHO) reported a rise in iGAS cases, including fatalities, in its European member states [10]. iGAS became a nationally notifiable disease in Australia on 1st July 2021 [11, 12]. However, Queensland and Northern Territory had started notifying iGAS in 2005 and 2011, respectively [12]. The incidence of iGAS varies significantly across different regions in Australia, likely due to differences in population dynamics, comorbidities, socioeconomic status, and climate [13, 14]. Longitudinal studies conducted in Australia revealed an increasing incidence of iGAS over time [12, 15–17]. Despite extensive research, GAS remains an unmet public health problem [18]. To the best of our knowledge, there has not been a systematic review and meta-analysis of the Australian epidemiology of iGAS. Therefore, we set out to estimate the burden of iGAS in Australia and describe the risk factors and outcomes of iGAS in different regions and population groups, through a systematic review and meta-analysis.

## Methods

### Search strategy and selection criteria

We searched for studies on iGAS published in MEDLINE, CINAHL, Scopus, Web of Science, Emcare and Pubmed databases. The final search was conducted on 25 October 2025. Full search terms are provided in Appendix 1. The search was restricted to published studies in humans. If information reported in the studies were not clear, the corresponding authors were contacted for further information. The review was registered in PROSPERO (CRD42023493112). The definition of iGAS of the included studies was either based on microbiological confirmation (e.g., *S. pyogenes* isolated from a sterile site) or identification using ICD-10 hospital discharge codes.

**Fig. 1:**
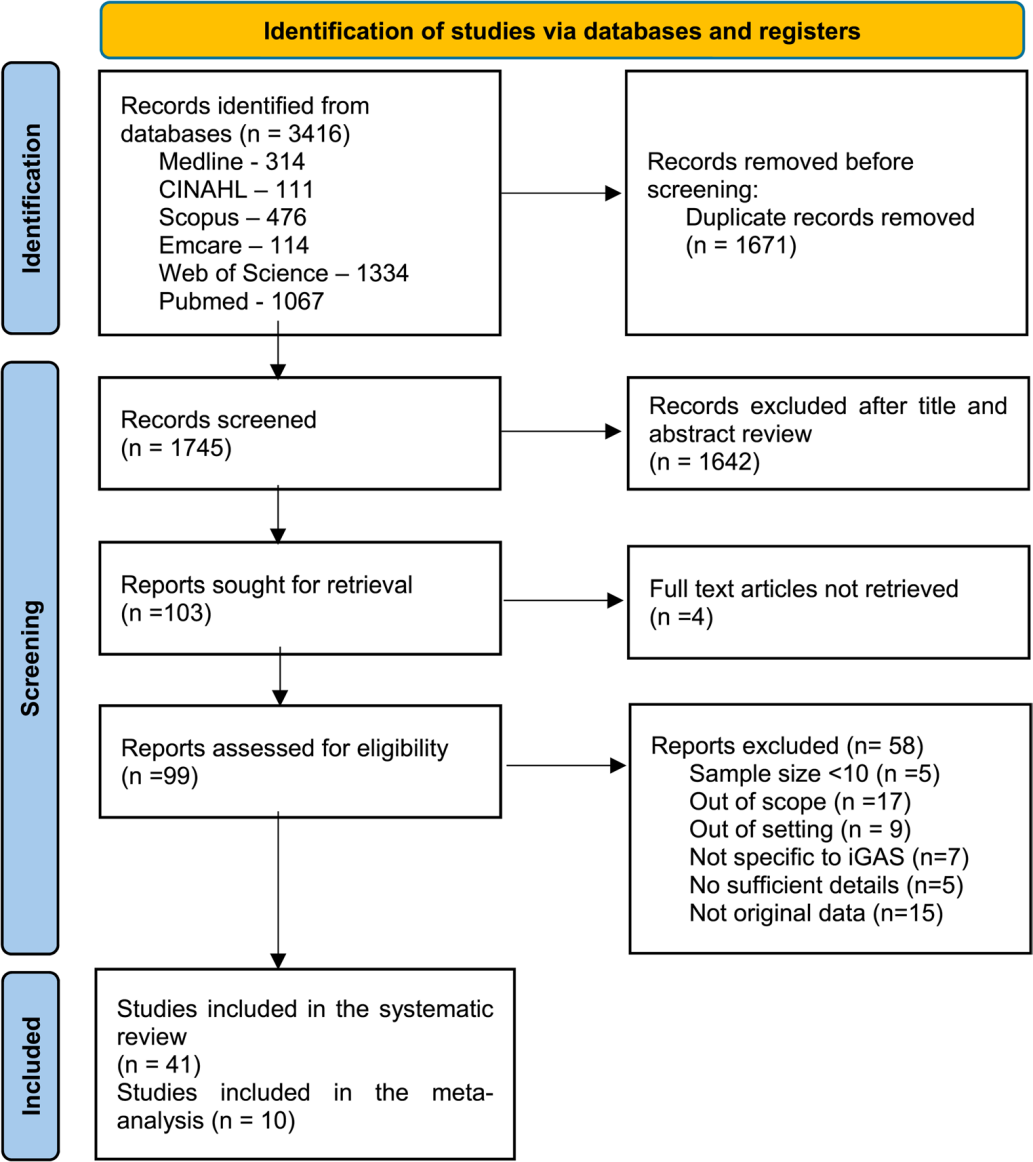
Data extraction for the systematic review

Two researchers (HR and EM) developed the search strategies after consultation with the liaison librarian. EndNote software was used to manage the searches. One reviewer (HR) conducted the de-duplication and the first screen by title and abstract. Two reviewers (HR and EM) then conducted a second screen, and disagreements were resolved with three other reviewers (DE, OA and AP). Full texts of the articles were retrieved on the second screen and reviewed by two researchers independently (HR, EM, DE, OA, AP) to finalise the studies to be included. Eight researchers (HR, EM, DE, OA, AP, CA, AT, GR) were involved in data extraction, and two researchers independently extracted data from each study. When duplicate data were identified (i.e., the same population and study period reported across multiple publications), we included the study providing the most complete information in the meta-analysis. Any discrepancies in extracted data were resolved through discussion among all researchers until consensus was reached. Studies conducted in the Northern Territory and Queensland were classified as tropical. For Western Australia, only studies that explicitly identified their setting as tropical or temperate were included in the meta-analysis, as only the northern region of Western Australia falls within the tropical zone (Fig. 1 in Appendix 2). The methodological quality of each study was rated by two reviewers independently using a nine-item quality assessment tool for studies reporting prevalence data adapted from The Joanna Briggs Institute (Appendix 3).

### Data analysis

We used the Preferred Reporting Items for Systematic Reviews and Meta-Analyses (PRISMA) to report included and excluded studies (Fig. 1). Studies that reported both the number of cases and the corresponding study population were included in the meta-analysis. In contrast, studies lacking clear information on the study population or setting, as well as those focusing solely on a single disease manifestation of iGAS, were included only in the systematic review. When studies reported incidence based on annual admissions or bed days, these were converted to cases per 100,000 person-years using population data from the Australian Bureau of Statistics (ABS). The pooled incidence of iGAS, along with its 95% confidence interval (CI), was calculated across the studies and for specific subcategories. The incidence of iGAS in tropical Australia was assessed using studies from purely tropical regions, as well as studies from Queensland and Western Australia if provided data on the proportion of participants from tropical areas. We used the meta and metafor packages in R version 4.0.3. Heterogeneity among studies was quantified using *I*^2^ statistics.

## Results

We identified 3416 published articles for consideration. Following removal at multiple stages, 41 articles were included in this review, of which 10 were included in the meta-analysis (Fig. 1). Characteristics of the studies included in this review are summarised in Table 1.

**Table 1:** Characteristics of Studies Included in the Review

Study	Study period	Study setting	Study population	Characteristics of study participants	Main study findings	Included in Systematic review or Meta-analysis
[19]	1976–1994	RDH, NT	191 cases of septic arthritis	Mean age is 30.2 years (6 months to 86 years) Males-63% Females-37%	31(16%) cases of septic arthritis due to GAS	Systematic Review
[20]	1996–2009	Townsville Hospital, QLD	392 episodes of bacteraemia caused by beta-haemolytic streptococci	Mean age-45.04 years (SD 25.38) Males-46.60% Females-53.40% Indigenous-52.88% Non-Indigenous-43.98%	191(49%) GAS bacteraemia cases. Incidence of GAS bacteraemia is 7.4/100,000/year* Crude mortality rate of GAS bacteraemia is 7.9% (CI 4.5, 12.6)	Systematic Review
[21]	2000–2009	Townsville Hospital, QLD	4536 significant bacteraemias	All age groups	136(3%) GAS bacteraemia cases	Systematic Review
[22]	2007–2009	13 major tertiary paediatric hospitals in all states and territories	174 children with Empyema	Median age-2.1 years (range 0.4–5.3 years)	3(2%) bacteraemias and 14(8.8%) empyema due to GAS	Systematic Review
[23]	2014–2018	Alice Springs Hospital, NT	662 Participants 799 bacteraemia episodes	Patients > 15 years of age Mean age-52 years Indigenous-541(82%) non-Indigenous-121(18%)	77(12%) GAS bacteraemia episodes Incidence of GAS bacteraemia is 106/100,000/year	Systematic Review
[24]	1998–2009	All major hospital laboratories in NT	295 confirmed cases of GAS bacteraemia	Mean (SD) age of 42.1 (22) years	Crude incidence of GAS bacteraemia is 15.8 (14.1–17.7)/100,000/year Age-adjusted incidence is 15.2 (13.4–16.9)/100 000/year	Systematic Review
[25]	1991–1996	RDH, NT	72 episodes GAS bacteraemia	Mean age-41.7 years	Crude incidence of GAS bacteraemia is 9.3/100,000/year	Systematic Review
[26]	2008–2019	Hunter New England Local Health District, NSW	486 GAS bacteraemia cases	Median age-61 years (IQR: 39–78 years)	Age-standardised incidence of GAS bacteraemia is 4.05/100,000/year Crude incidence of GAS bacteraemia is 4.53/100,000/year	Systematic Review
[27]	2014–2017	A tertiary hospital in Melbourne, Victoria	43 GAS bacteraemia cases	Average age-52 years (range 15–88 years)	All-cause mortality is 5%. Septic shock-12(28%) Intensive care unit admission-11(26%) surgical intervention-13(30%)	Systematic Review
[28]	1982–1993	RCH, Melbourne, Victoria	42 GAS bacteraemia cases	Age 1 month to 20 years	There was an increased incidence of infections (from 47% to 80%), and an increase in their severity, during the study period.	Systematic Review
[29]	2006–2020	SA	167 cases of necrotising soft tissue fasciitis	Median age 52 years (IQR:45–65)	23(36%) of the monomicrobial infections and 13(5.7%) of the polymicrobial infections were due to GAS.	Systematic Review
[30]	July 2016 to June 2018	7 Major hospitals (CHW, RCH, WCH, PCH, LCCH, RDH, MCH)	181 iGAS cases	Median age of 2.9 years (7 days to 16 years)	Case fatality rate-5(2.8%) Severe disease-74(40.9%)	Systematic Review
[31]	2019	5 Major hospitals (RCH, MH, QCH, PCH and RDH) in Australia	48 iGAS cases	Paediatric patients	No other information mentioned on the annual report	Systematic Review
[32]	July to December, 2016	6 Major hospitals (CHW, RCH, WCH, PMH, LCCH and RDH)	45 iGAS cases	Paediatric patients	ICU admission-19(42%) Severe disease-8(18%)	Systematic Review
[33]	2001–2010	North QLD	696 positive blood cultures	Children < 16 years of age	18 (2.6%) GAS bacteraemia. Stable trend of GAS bacteraemia over the period.	Systematic Review
[34]	2011–2018	CHW, Sydney	192 cases of empyema	Median age-3.0 years (IQ:1-5yrs)	13 GAS empyema cases	Systematic Review
[35]	2014–2016	RCH, Melbourne	28 iGAS cases	Median age-3.5 years (4 days to 11 years)	*Incidence of iGAS among paediatric population is 1.29/100,000/year STSS-5(18%), Pneumonia-6(21%), Osteoarticular-5(18%), Cellulitis-5(18%), NF-1(4%)	Systematic Review
[36]	1996–2004	QLD	659 sterile site isolates from laboratories around Queensland.	Median age 41 years (3 weeks to 99 years)	Fatality rate-13.5%. All isolates were sensitive to penicillin and vancomycin.	Systematic Review
[37]	2007–2008	RDH, NT	48 episodes of GAS sepsis	Mean age 46.7 years.	12(6.2%) bacteraemias, 35(8.7%) of community acquired sepsis and 13 (9.5%) of Health care associated sepsis due to GAS	Systematic Review
[38]	2007–2018	Two tertiary referral hospitals in Newcastle	13 cases of GAS community acquired pneumonia	Mean age-48 years (18–77 years)	Mortality rate-2 (15%) ICU admissions-12(92%)	Systematic Review
[39]	1994–2001	RDH, NT	14 cases of GAS NF	Mean age 41.5 years	Mortality rate-4 (29%) ICU admissions-13(93%)	Systematic Review
[40]	1996–2005	North QLD	25 patients who fulfilled criteria for STSS and 31 matched controls with invasive GAS disease without evidence of STSS	Mean age- -cases-45.3 years -controls-44.8 years	Mortality was significantly higher (28%) in the STSS group. Necrotising fasciitis was significantly associated with STSS.	Systematic Review
[41]	2003–2014	RCH Melbourne and Monash Medical Centre, Victoria	19 cases of STSS	< 18 years of age Median age-5 years (IQR:3.8–8.9)	Mortality rate − 0 ICU admissions − 11(58%)	Systematic Review
[42]	1999–2019	RDH, NT	504 episodes of GAS bacteraemia		Significant increase in incidence from 12.2 episodes/100,000/year in 1999 to 20.4/100,000/year in 2019 (*p* = 0.006).	Systematic Review
[43]	2014–2020	Far North QLD	286 episodes of GAS bacteraemia	Median (IQR) age-60 (48–71) years. 169(59.1%) Indigenous Australian.	Mean annual incidence in the total population was 14.5/100,000/year and 49.2/100,000/year among the Indigenous population.	Systematic Review
[44]	2010–2013	RCH Melbourne	12 cases of iGAS	Median age (IQR) -6 (2.5-8) years	Pneumonia = 5 cases, bacteraemia = 4 cases, septic arthritis = 3 cases. Nine patients had multi-organ failure.	Systematic Review
[45]	2016–2018	7 major paediatric hospitals in Victoria, WA, NSW, QLD, SA and NT	181 lab confirmed cases of iGAS	Children ≤ 18 years (7 days to 16 years)	Mean annual minimum incidence rate was 1.6/100,000 children/year (95% CI:1.1, 2.3)	Systematic Review only
[46]	2008 and 2010	Western Sydney, Australia	72 patients with iGAS-pathology data 2008-25 2010-47	All age groups Median (IQR)- 2008-45 years (38–50) 2010-40 years (26–64)	*(2.9/100000/year in 2008 and in 5.4/100000/year in 2010)-calculated using denominator and case number in the article.	2008 data - Both 2010 data – Systematic review only as the study reported an outbreak
[47]	2007–2017 (11 years)	Laboratory results reported to the Victorian Hospital Pathogen Surveillance Scheme (VHPSS)	1,979 cases of iGAS (1,369 confirmed and 610 probable cases)- analysed both confirmed and probable	Males-1,073(54.2%) Females-906(45.8%) Among the 1,744 cases with hospitalisation data, 23 (1.3%) were Indigenous.	Incidence of iGAS is 3.1/100,000/year in 2007. Incidence of iGAS is 5.2/100,000/year in 2017. Incidence among the Indigenous is 3.9/100,000/year. Median annual incidence is also 3.1	Both
[48]	2007–2017	Victoria	1976 cases of iGAS	Total population	** Mean annual incidence in the total population was 3.6/100,000/year	Systematic review only
[49]	2007–2017	Victoria	1,311confirmed iGAS cases	All ages	Mean annual incidence is 2.1/100,000/year	Systematic review only
[50]	2002–2004	Victoria	333 confirmed iGAS cases	All age groups Mean age-45.8 years (95% CI:42.6–49.0 years)	Average Incidence of iGAS 2.7 (95% CI, 2.3, 3.2) /100,000/year. Incidence among Indigenous population is 2.9/100,000/year.	Both
[12]	January 2023-June 2023	Whole Australia	1185 confirmed iGAS cases-from National Notifiable diseases surveillance system	All age groups	Incidence of iGAS is 9/100,000/year	Both
[51]	2000-2018-19 years	WA (all WA public and private hospital records)	2237 iGAS cases 0.87% lab confirmed and 29% from ICD-10 codes (56% of this 29% was also lab confirmed).	All age groups. Median age-44 years	*Incidence of iGAS for the total population is 4.53/100,000/year **Incidence of iGAS among the Indigenous population is 60/100,000/year	Both
[52]	2007 and 2008	Whole QLD (data from QLD health).	67 cases of iGAS	Patients aged 0–18 years	Incidence of iGAS 3.5 and 2.6/100,000 children/year in 2008 and 2007 for the total population. Incidence of iGAS among the Indigenous population 13.2 and 9.9/100,000 children/year in 2008 and 2007.	Both
[53]	1996–2001	North QLD	109 iGAS cases	The median age is 39 years (1 month − 88 years)	Incidence of iGAS among the Indigenous population is 82.5/100,000/year compared with 10.3/100,000/year in the non-indigenous patients. *Incidence for the total population 14.27/100,000/year	Both
[54]	2011–2021	NT (Notifiable Diseases System)	692 confirmed iGAS cases	Median age-50(IQR: 37–63 years) 133 cases of iGAS receiving dialysis.	The age-standardised incidence of iGAS in very remote areas-57.1/100,000/year remote areas-40.9/100,000/year outer regional areas-15.7/100,000/year Crude incidence-34.3/100,000/year Incidence among Indigenous-106/100,000/year	Both
[13]	2011–2013	NT	128 episodes of iGAS disease occurred in 121 people.	Indigenous-99(77%) Non-Indigenous-29(23%) Haemodialysis patients-21(16%)	Incidence of iGAS is 28/100,000/year. Incidence among Indigenous is 70/100,000/year. Incidence among non-Indigenous is 8·8/100,000/year. Incidence among Haemodialysis patients is 2205·9/100,000/year.	Systematic review only
[15]	2018–2022	5 paediatric hospitals in Victoria, QLD, WA, NT	280 cases of iGAS (PEADS network. Pathology confirmed or met clinical criteria)	Less than 18 years. median age 4.5 years (interquartile range 1.4–6.4)	Mean annual incidence of iGAS across the study period was 1.8 per 100,000 children (95% CI 1.7, 1.9)	Both
[55]	2014–2020 (7 Years)	Cairns Hospital-Far North Queensland	286 episodes of GAS bacteraemia cases occurred in 274 individuals.	Median (IQR) age was 60 (48–71) years	In multivariable analysis, systolic blood pressure < 100 mmHg (*p <* 0.0001), serum lactate > 4 mmol/L (*=* 0.001), a circulating lymphocyte count < 0.5 × 10^9^/L (*p =* 0.02) and a serum albumin < 30 g/L (*p=* 0.049) were independent predictors of death or ICU admission. STSS and/or necrotising fasciitis were significantly associated with death *p* < 0.0001).	Systematic review only
[56]	2011–2023 (12 Years)	Five tertiary or quaternary health networks - Two in Melbourne (VIC) and two in Sydney (NSW) and one in the top end of the Northern Territory	Laboratory confirmed cases. NT − 500 iGAS cases VIC and NSW – 495 iGAS cases	Mean age (SD) years NT − 53 (37–64) SE Australia − 53 (35–70)	The crude IRR for the Top End compared with southeast Australia was 5·97 (95% CI 4·61–7·73). The odds of in-hospital mortality (OR 0·43, 95% CI 0·26–0·70), 30-day mortality (0·38, 0·23–0·63), and ICU admission (0·42, 0·30–0·59) were lower in the Top End than in southeast Australia. Average annual incidence = 23.1/100,000/year**	Both

* Population mentioned on the article was used for the calculation of incidence

** Population data from the Australian Bureau of Statistics was used for the calculation of incidence

*NSW* New South Wales, *WA* Western Australia, *QLD* Queensland, *SA* South Australia, *NT* Northern Territory

*RCH* The Royal Children’s Hospital, Melbourne, Victoria, *MH* Monash Health, Melbourne, Victoria, *QCH* Queensland Children’s Hospital, Brisbane, Queensland, *PCH* Perth Children’s Hospital, Perth, Western Australia, *RDH* Royal Darwin Hospital, Darwin, Northern Territory, *CHW * The Children’s Hospital at Westmead, Sydney, New South Wales, *WCH* Women’s and Children’s Hospital, Adelaide, South Australia, *LCCH* Lady Cilento Children’s Hospital, Brisbane, Queensland

### Systematic review of the studies

#### Focus of infection and presentations of iGAS

SSTI was the commonest focus of infection (35.6–71.3%) [24, 25, 43, 46, 47, 53, 56]. iGAS was most commonly isolated from blood (72–94% of cases) [50, 53]. Paediatric studies reported that around 70% of iGAS cases were bacteraemia [30, 35, 45]. In Queensland, Northern Territory and Central Australia, GAS accounted for 3–12% of all reported bacteraemias [21, 23, 42]. GAS was responsible for approximately 2.6% of all bacteraemias among children [33]. The overall incidence of GAS bacteraemia in Australia ranged between 2 and 20.4 per 100,000 person-years [21, 25, 42, 50]. The incidence of GAS bacteraemia was significantly rising in the Northern Territory [42]. Other common presentations were pneumonia (10–25%), bone and joint infections (8–18%), post partum sepsis (8.5%) and meningitis (3%) [25, 35, 46, 53, 57].

#### Severe outcomes of iGAS

Several studies documented high mortality and significant rates of severe disease due to iGAS [47, 50, 51]. Around 25–36% iGAS cases were defined as severe iGAS disease [54, 58]. Severe disease rates among children was even higher, 36–41% [30, 35]. Around 6.6–16.5% of iGAS was due to STSS [13, 24, 25, 43, 45, 50, 53]. A study conducted in Victoria showed that, 28% of GAS bacteraemia cases led to septic shock [27]. Further, around 80% of GAS bacteraemias led to sepsis, while 47% led to severe sepsis [24]. STSS among paediatric patients with iGAS was around 6–18% [30, 35, 52]. The reviewed studies revealed that the rate of NF was 0.26-11% [24, 25, 47, 50]. A paediatric study conducted in Victoria found that 4% of NF cases in children were caused by GAS [35]. 

The rate of ICU admission among patients with iGAS ranged from 6 to 33% across all ages [13, 24, 26, 27, 43, 46, 47, 50, 51, 58], and from 14 to 46% among children [30, 35, 45, 52]. The ICU admission rate for STSS was higher at 58% [41]. Reported mortality due to iGAS ranged from 5.6 to 13.8% [13, 20, 24–26, 36, 43, 46, 47, 50, 53, 58] while mortality among children was lower, ranging from 2.8% to 4% (2.8–4%) [30, 45, 52]. Mortality associated with STSS was considerably higher than that observed for other iGAS presentations, ranging from 22% to 28% [40, 53].

#### Antimicrobial resistance

Penicillin remains the first-line treatment for iGAS, while macrolides or lincosamides are recommended for patients with penicillin allergy. Tetracyclines and fluoroquinolones may serve as alternative agents in selected cases. In 2019, *Streptococcus pyogenes* was ranked 16th among 23 pathogens assessed in a systematic review evaluating the global burden of antimicrobial resistance [59]. Limited data are available regarding antimicrobial resistance patterns in iGAS specifically. Australian studies have reported resistance to erythromycin (2.7–3.4%) and tetracycline (11–16.8%), with no documented resistance to penicillin [24, 36, 43, 53]. However, detailed information on the clinical and demographic characteristics of patients with resistant isolates remains sparse.

#### Risk factors for disease and risk factors for severe outcomes

##### Age and sex

Epidemiological studies consistently demonstrated a bimodal age distribution of iGAS, with the highest incidence observed in children aged < 5 years and in older adults, especially those aged ≥ 60–75 years [24, 26, 47]. Older age also being associated with increased mortality and ICU admission [26, 51]. The association between iGAS and sex has been less consistent. While some studies reported a higher incidence among males [60] others found a greater incidence in females [54]. Furthermore, several studies suggested that the association with sex varied across age groups [49]. In contrast, a paediatric study reported no significant association between disease severity and either age or sex [30].

##### Chronic diseases and behavioural risk factors

Chronic diseases were common among patients with iGAS. In Far North Queensland and the Northern Territory, 66–79% of patients had underlying chronic conditions [13, 43] while a study conducted in Queensland revealed that 17% of children with iGAS had a chronic condition [52]. A Victorian study reported that renal failure and cardiac diseases were frequently observed as risk factors for iGAS [47]. Another study conducted in far North Queensland revealed that diabetes was significantly associated with death or ICU admission (*p* < 0.05) although this was not significant in the multivariable analysis [55]. In their study, Sivagnanam et al., (2015) showed that factors significantly associated with mortality due to iGAS included Acute Physiology and Chronic Health Evaluation II (APACHE II) score > 30, bacteraemia without a focus, acute renal failure and acute respiratory distress syndrome [46]. In this study, the incidence of iGAS among patients receiving haemodialysis was very high (1643/100,000 per year) [54]. Elsewhere, higher Sequential Organ Failure Assessment (SOFA), STSS, severe sepsis and the receipt of intravenous immunoglobulin and haemodialysis were found to be significantly associated with mortality due to iGAS [24]. Behavioural risk factors were also noted: injecting drug use was reported in up to 42% of cases of GAS bacteraemia in Victoria [27] and hazardous alcohol use (defined as documented evidence in the medical records in the 12 months prior to presentation) was found to be associated with increased ICU admission or death due to iGAS [55]. Hazardous drinking is defined as a quantity or pattern of alcohol consumption that places individuals at risk for adverse health events by WHO [61]. 

##### Indigenous status

iGAS was commoner in the Indigenous population [20, 24, 26, 54, 56]. In addition, the mean ages of people with iGAS bacteraemia and of those who died were each lower for Aboriginal than non-Aboriginal people [23]. However, some studies revealed no significant difference in the incidence of iGAS between Indigenous and non-Indigenous populations [47]. Indigenous status was not associated with increased mortality, STSS or ICU admission according to studies conducted in Northern Territory, Queensland and Western Australia [13, 43, 51]. There was no association between disease severity and Indigenous status according to a paediatric study as well [30].

### Meta-analysis results

**Fig. 2:**
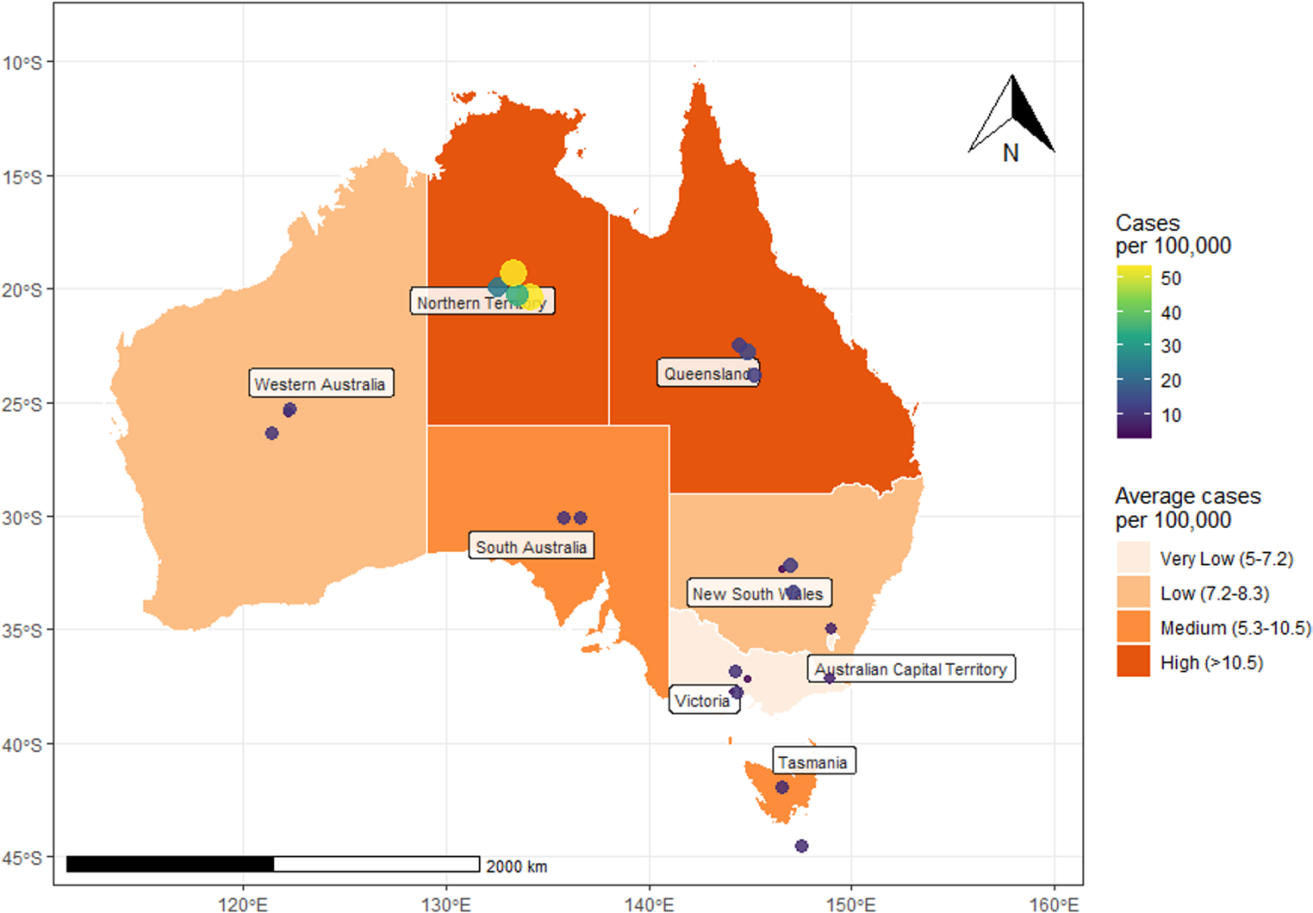
Reported burden of iGAS across Australia states and territories. Coloured circles represent the incidence of iGAS as reported by the studies and the orange colour scheme shows the average cases per 100,000 person-years for states and territories based on the study results

**Fig. 3:**
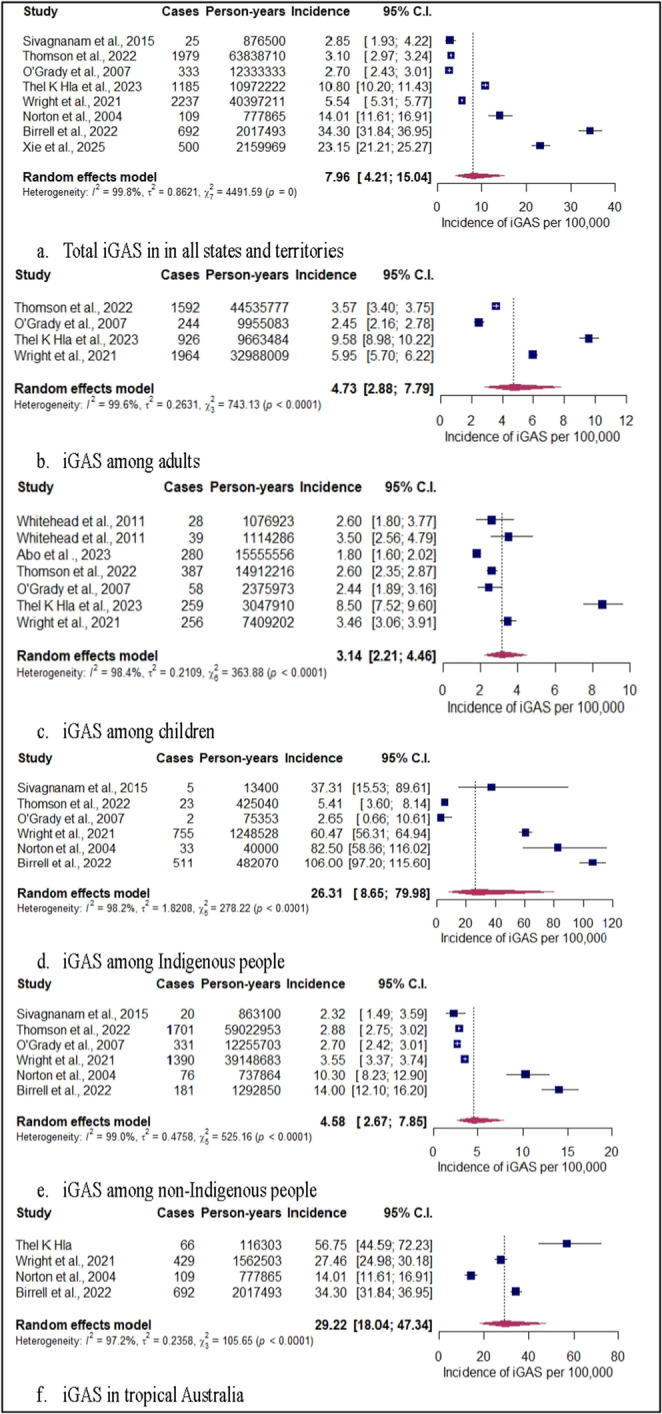
Meta analysis of the Incidence of iGAS in Australia

The meta-analysis included studies from all Australian states and territories across all age groups from 1996 to 2023. Figure 2 depicts the incidences as reported in the studies and the average burden of iGAS across states and territories in Australia. Accordingly, Northern Territory and Queensland had the highest disease burden. We conducted meta-analysis for 10 studies estimating the pool incidence across all studies as well as on several subcategories (paediatric, adults, Indigenous, non-Indigenous and population in tropical regions) (Fig. 3). We calculated the pooled incidence of iGAS to be 7.96 (95% CI: 4.21–15.04) per 100,000 person-years. There was not much difference in the incidence between adults (4.73, 95% CI [2.88–7.8] per 100,000 person-years) and children (3.14 [95% CI: 2.21–4.46] per 100,000 person-years). Indigenous patients had a very high incidence of 26.3 (95% CI: 8.65–79.98) per 100,000 person-years compared to non-Indigenous: 4.58 (95% CI: 2.67–7.85) per 100,000 person-years. The tropical subcategory had an incidence of 29.22 (95% CI: 18.04–47.34) per 100,000 person-years. Heterogeneity was high in all meta- analyses (I²=99.8–97.2%).

### Analysis of emm-types

We also characterized the distribution of *emm* types by state and territory across Australia. *Emm* typing is a widely utilized molecular tool for epidemiological surveillance, vaccine development, and outbreak investigations. The M protein, encoded by the *emm* gene, is a key virulence factor of *Streptococcus pyogenes*, responsible for inducing type-specific antibody responses in humans. Understanding the molecular epidemiology of GAS within a given region is essential for assessing potential vaccine coverage. The predominant *emm* types identified nationally were *emm*1, *emm*89, *emm*12, and *emm*4, findings that are consistent with previous paediatric studies [15, 45]. A detailed breakdown of *emm* type distribution by state and territory is provided in Appendix 4.

A few studies have reported that *emm1* is associated with mortality and severe disease [35, 50]. A paediatric study conducted in Queensland found that emm4 was not associated with severe disease [62]. In addition to the commonly identified *emm* types, a study conducted in Victoria reported that six types were found only in people who inject drugs (PWID). In this study, *emm101* was identified in three cases (four cases including emm101.1) [27]. A study conducted in NSW by Sivagnanam et al. reported that there were no significant differences in *emm* types or clusters between cases with fatal and non-fatal outcomes [63].

## Discussion

### Disease incidence

Our systematic review and meta-analysis revealed a significant burden of iGAS across Australia, with a pooled incidence of 7.96 per 100,000 person-years. However, in our subgroup analysis, we observed substantial variation in incidence, influenced by factors such as age, Indigenous status, and climate zone. The highest incidence rates were observed among the Indigenous population and those living in tropical regions of Australia. Tropical Australia is home to approximately 6.4% of the Indigenous population, compared to 2.9% in the temperate regions (excluding Western Australia) [64]. Our subgroup analysis showed that the Indigenous people in tropical regions had the highest burden. By comparison, the incidence of iGAS in developed countries ranged from 3 to 4 per 100,000 person-years, while developing countries and disadvantaged communities within developed nations experienced significantly higher rates, ranging from 11 to 80 per 100,000 per year, highlighting global disparities in iGAS incidence [60, 65].

### Focus of infection and common presentations of iGAS

Consistent with our findings, SSTI was the most common primary focus of iGAS in many studies worldwide [66–68]. The current study revealed that more than 70% iGAS were bacteraemic. Similarly, European studies have reported bacteraemia in 66–78% of iGAS cases with 19–25% of cases presenting as bacteraemia without a focal source [69–72]. A Canadian study also identified primary bacteraemia as the most common clinical manifestation of iGAS, accounting for 33% of cases [73]. In the present review, GAS accounted for 3–12% of all bacteraemia cases in Australia across all age groups and 2.6% among paediatric cases. In comparison, an Israeli study reported that GAS represented 0.6% of adult bacteraemia and 3.3% of paediatric bacteraemia [74). The high rates of iGAS and the association with skin infection highlight that preventing secondary bacterial infections primarily rely on effectively treating the underlying skin condition. Additionally, educating patients about maintaining proper skin hygiene and promptly reporting any changes in their primary lesions is essential.

### Disease trend and seasonality

Studies in this review had varying disease trends. Yet, it is important to highlight that there was considerable heterogeneity among the studies, which may have influenced the detection of any existing trend if indeed one exists. The studies conducted in the Northern Territory accounted for the majority of the heterogeneity observed in the data, suggesting that regional factors may have played a significant role in the variability of the findings.

The reviewed studies showed a seasonal pattern of high incidence during the winter season, although it was not consistent across all the studies. Similarly, several studies conducted in other countries revealed increased cases in winter and early spring [3, 16, 65, 75]. However, a study conducted in the USA revealed that some strains peak in winter while some peak in summer depending on the *emm* type of the bacteria strain [76]. Although seasonality is prominent in temperate countries, it is less visible in tropical regions [77]. In our meta-analysis tropical studies showed a high incidence compared to studies conducted in temperate settings. The probable reasons for this could be that the warm, humid conditions in tropical regions promote skin barrier breakdown due to sweating, insect bites and minor trauma leading to bacterial survival on the skin. Other reasons could be the population structure, access to healthcare and hygiene and other social factors, as most tropical areas are remote or regional. However, a study conducted in England reported an out-of-season surge, which the authors attributed to increased susceptibility to infections resulting from lockdown measures during the COVID-19 pandemic [78]. In addition, some studies showed an epidemiological correlation of iGAS with seasonal influenza [6, 45, 60].

### Risk factors for disease and risk factors for severe outcomes

According to this systematic review, very young age, old age, presence of chronic diseases, IV drug use, Indigenous status and living in a tropical region were among the risk factors for iGAS while association with sex was mixed. Although the included studies did not provide sufficient detail, it would be important to determine whether the higher prevalence observed among females may be attributable to the inclusion of women of childbearing age, particularly in relation to puerperal sepsis. In addition, the presence of severe sepsis, admission to ICU, receipt of intravenous immunoglobulin and haemodialysis were associated with severe outcomes. Studies outside Australia reported that male sex is a risk factor for GAS, likely due to higher rates of chronic diseases, intravenous drug use and alcoholism in males [79, 80]. Given the smaller number of studies in our meta-analysis it was not possible to conduct a meta-regression.

According to studies conducted outside of Australia, elderly and very young populations were at higher risk of acquiring iGAS while mortality was associated with increasing age [3, 65]. Diabetes, heart disease and smoking were found to be probable risk factors for invasive disease [3]. Increasing age, residence in a nursing home, recent surgery, presence of specific syndromes (septic shock, NF, meningitis, isolated bacteraemia, or pneumonia), underlying chronic illness or immunosuppression were found to be predictors of death according to a US study which used data from Centers for Disease Control and Prevention [3]. A scoping review on paediatric iGAS revealed that varicella infection, chronic illnesses and low household income were risk factors for iGAS [81]. Apart from these, HIV infection and intravenous drug use were also reported as risk factors [65, 82, 83].

Studies have consistently shown high iGAS incidence rates among Indigenous communities in countries like the USA and Canada, linked to population dynamics and individual risk factors [84, 85]. According to the meta-analysis of the present study, the pooled incidence of iGAS among the Indigenous was about 7 times higher than that among the non- Indigenous. Similarly, a Canadian study revealed a ten times higher incidence rate among the First Nations communities compared to the provincial and national averages [85]. A recent national study conducted in New Zealand also revealed comparable disparities, with Pacific and Māori populations experiencing the higher iGAS incidence rates [86].

Enhancements in living standards, control and management of chronic diseases and health education to high-risk groups are likely to contribute to reducing disease rates. At the same time, it is important to consider targeted clinical strategies to reduce transmission. One such strategy is the administration of prophylactic antibiotics to high-risk close contacts. Although this approach appears promising, and some Australian guidelines and experts recommend antibiotic chemoprophylaxis to reduce the risk of invasive iGAS infection among household contacts [87, 88] the current evidence supporting this practice remains limited, and the available data are not sufficient to support widespread implementation [89, 90].

### Severe outcomes of iGAS

According to our review, mortality due to iGAS was between 5.6 and 13.8%. Mortality rates differed markedly between developed and less developed countries. Mortality rates of iGAS in Europe and the USA ranged between 14% and 19% [66, 91–95]. Although GAS is more prevalent in low-income countries, limited data exists due to a lack of information system and research [5, 18, 60]. Many studies from developing countries have reported mortality rates that were several times higher than in developed countries [5, 60]. A mortality rate of around 25% was reported from studies conducted in Fiji and Kenya [96–98]. These higher mortality rates may reflect delayed access to healthcare, limited availability of advanced medical interventions, higher prevalence of comorbidities and under resourced health systems.

The current review revealed that up to 16.5% iGAS were STSS while up to 11% developed NF. According to European studies, iGAS lead to NF in 8% of cases [66] and STSS in 13–15% [99, 100]. We found that the CFR of STSS in Australia was around 22–28%. As some studies reported mortality in different units, it was difficult to compare the mortality rates across all studies. Several studies outside Australia reported a high CFR of 24–50% for NF(95, 101, 102) and 36–50% for STSS [95, 99, 103]. NF and STSS are severe manifestations of iGAS, requiring clinicians to suspect and promptly refer for surgical evaluation if needed.

The present review included studies revealing resistance to macrolides and tetracycline. Similar findings were reported from Africa, Europe and USA [93, 104–108]. The rate of antibiotic resistance of these studies ranged between 1 and 76% for macrolides [93, 104–108] and 8 to 15% for tetracycline [93, 104, 109]. Similar to the present review, two studies from USA and Norway reported a 100% susceptibility to beta-lactams [105, 109]. Antibiotic resistance is more prevalent among homeless people and individuals with a history of intravenous drug use [105, 106] likely reflecting increased exposure to antibiotics, frequent healthcare encounters and higher prevalence of colonisation with resistant strains in these populations. This emphasises the importance of ongoing surveillance and antimicrobial susceptibility testing to track the trends of increasing antimicrobial resistance.

### *emm*-types

Although emm types 1, 89, 12, and 4 have been identified as highly prevalent in Australia, no consistent pattern of distribution was observed, reflecting the overall genetic diversity of circulating GAS strains. Notably, these same *emm* types have also been reported as predominant in a review conducted in Europe and North America, indicating their widespread global circulation [110]. The M protein, encoded by the *emm* gene, is a major virulence factor and plays a key role in eliciting type-specific antibody responses in humans. However, the association between specific *emm* types and their pathogenic potential, including their relationship to disease severity, remains controversial and is not yet fully understood [46, 52]. Future studies should aim to provide a comprehensive analysis of *emm* type distribution stratified by age, sex, clinical presentation, seasonality, geographic region, antimicrobial resistance patterns, and mortality outcomes. This would inform targeted prevention strategies, vaccine development, and public health policy.

### Limitations

The studies included in this review varied considerably in terms of sample size, population characteristics, and geographical setting. Despite our efforts to conduct analyses within subcategories to reduce heterogeneity, this also led to fewer studies per category, reducing the power to detect effects. This review used studies with varying definitions for iGAS that affect diagnostic specificity and completeness, potentially contributing to heterogeneity in reported incidence. Moreover, studies conducted in response to outbreaks may not accurately reflect the true prevalence of the condition compared to those conducted under normal, non-outbreak conditions. The absence of geographic information in some studies, specifically whether they were conducted in tropical or non-tropical environments, led to their exclusion from certain subcategory analyses. There was high heterogeneity between studies due to differing methodologies and varying characteristics of study participants. When the target population was not specified, ABS data was used to estimate the denominator population, potentially leading to over or under-estimation of disease incidence. As iGAS became a nationally notifiable disease in 2021 and different states and territories adhered to this regulation at different times, there may have been an underestimation of the number of reported cases.

## Conclusion

This study highlights the significant burden of iGAS infection in Australia especially in tropical settings and among Indigenous communities. Understanding the different risk factors for disease and outcomes can be more precisely made with large health cohort databases, hence analysis of linked electronic medical records may advance our understanding of disease burden, and lead to more efficient allocation of resources. The insights gained from this review can inform public health policies and strategies to mitigate the health and economic impacts of iGAS. Moreover, the wide variety of emm-types means that a successful GAS vaccine must cover many circulating strains.

## Supplementary Information

Below is the link to the electronic supplementary material.

Supplementary Material 1

## Data Availability

No datasets were generated or analysed during the current study.

## Abbreviations

ABS: Australian Bureau of Statistics
APACHE II: Acute Physiology and Chronic Health Evaluation II
CI: Confidence Interval
GAS: Group A *Streptococci*
iGAS: Invasive Group A *Streptococci*
NF: Necrotizing fasciitis
NSTI: Necrotizing soft tissue infections
NSW: New South Wales
NT: Northern Territory
PRISMA: Preferred Reporting Items for Systematic Reviews and Meta-Analyses
PWID: People who inject drugs
QLD: Queensland
SA: South Australia
SOFA: Sequential Organ Failure Assessment
SSTI: kin and soft-tissue infections
STSS: Streptococcal Toxic Shock Syndrome
WA: Western Australia
WHO: World Health Organization
