# Supplementary material for: Burden of invasive group a streptococcus infection in Australia: a systematic review and meta-analysis
Source: Eur J Clin Microbiol Infect Dis. 2026 Apr 10;45(7):1901–16. doi: 10.1007/s10096-026-05480-x (PMC13328315; doi:10.1007/s10096-026-05480-x)
Supplement: Supplementary file 1 — Supplementary Material 1 [file 10096_2026_5480_MOESM1_ESM.docx]

**Appendix 1**

**Search strategy for iGAS (MEDLINE, CINAHL, Scopus, Web of Science, Emcare and Pubmed)**

1. **Medline**

| **Last run via:** | **OVID** |
| --- | --- |
| **Search Screen:** | **Advanced Search** |
| **Date of last search** | 25/10/2025 |

| 1 | Streptococcus pyogenes/ |
| --- | --- |
| 2 | (group A strep* or strep* group A or strep* pyogenes).mp. [mp=title, book title, abstract, original title, name of substance word, subject heading word, floating sub-heading word, keyword heading word, organism supplementary concept word, protocol supplementary concept word, rare disease supplementary concept word, unique identifier, synonyms, population supplementary concept word, anatomy supplementary concept word] |
| 3 | exp Australia/ |
| 4 | australia*.mp. |
| 5 | "Australian Aboriginal and Torres Strait Islander Peoples"/ |
| 6 | ("Indigenous Australian*" or "Australian Aboriginal*" or "Torres Strait Islander*" or "Australian first nation*").mp. [mp=title, book title, abstract, original title, name of substance word, subject heading word, floating sub-heading word, keyword heading word, organism supplementary concept word, protocol supplementary concept word, rare disease supplementary concept word, unique identifier, synonyms, population supplementary concept word, anatomy supplementary concept word] |
| 7 | 1 or 2 |
| 8 | 3 or 4 or 5 or 6 |
| 9 | 7 and 8 |

1. **Emcare**

| **Last run via:** | **OVID** |
| --- | --- |
| **Search Screen:** | **Advanced Search** |
| **Date of last search** | 25/10/2025 |

| 1 | Streptococcus pyogenes/ |
| --- | --- |
| 2 | (Group A strep* or strep* group A or strep* pyogenes).mp. [mp=title, abstract, heading word, drug trade name, original title, device manufacturer, drug manufacturer, device trade name, keyword heading word] |
| 3 | australia/ or exp australian capital territory/ or exp new south wales/ or exp northern territory/ or exp queensland/ or exp south australia/ or exp tasmania/ or exp victoria/ or exp western australia/ |
| 4 | indigenous people/ or exp first nation/ or exp indigenous australian/ |
| 5 | ("Indigenous Australian*" or "Australian Aboriginal*" or "Torres Strait Islander*" or "Australian first nation*").mp. [mp=title, abstract, heading word, drug trade name, original title, device manufacturer, drug manufacturer, device trade name, keyword heading word] |
| 6 | australia*.mp. [mp=title, abstract, heading word, drug trade name, original title, device manufacturer, drug manufacturer, device trade name, keyword heading word] |
| 7 | 1 or 2 |
| 8 | 3 or 4 or 5 or 6 |
| 9 | 7 and 8 |

1. **CINAHL**

| **Last run via:** | EBSCOhost Research Databases |
| --- | --- |
| **Search Screen:** | **Advanced Search** |
| **Date of last search** | 25/10/2025 |

| S1 | "strep* group A" |
| --- | --- |
| S2 | "Group A strep*" |
| S3 | “strep* pyogenes" |
| S4 | (MH "Australia+") |
| S5 | "australia*" |

1. **Scopus, Web of Science and PubMed (search conducted on 25/10/2025)**

"streptococc* group A" OR "group A strep*" OR "streptococcus pyogenes"

AND

australia* OR aboriginal* OR "torres strait islander*" OR queensland* OR "New south wales" OR "NSW" OR victoria* OR tasmania* OR "northern territory” OR “western Australia” OR “southern Australia” OR “Australian capital territory”

**Appendix 2**


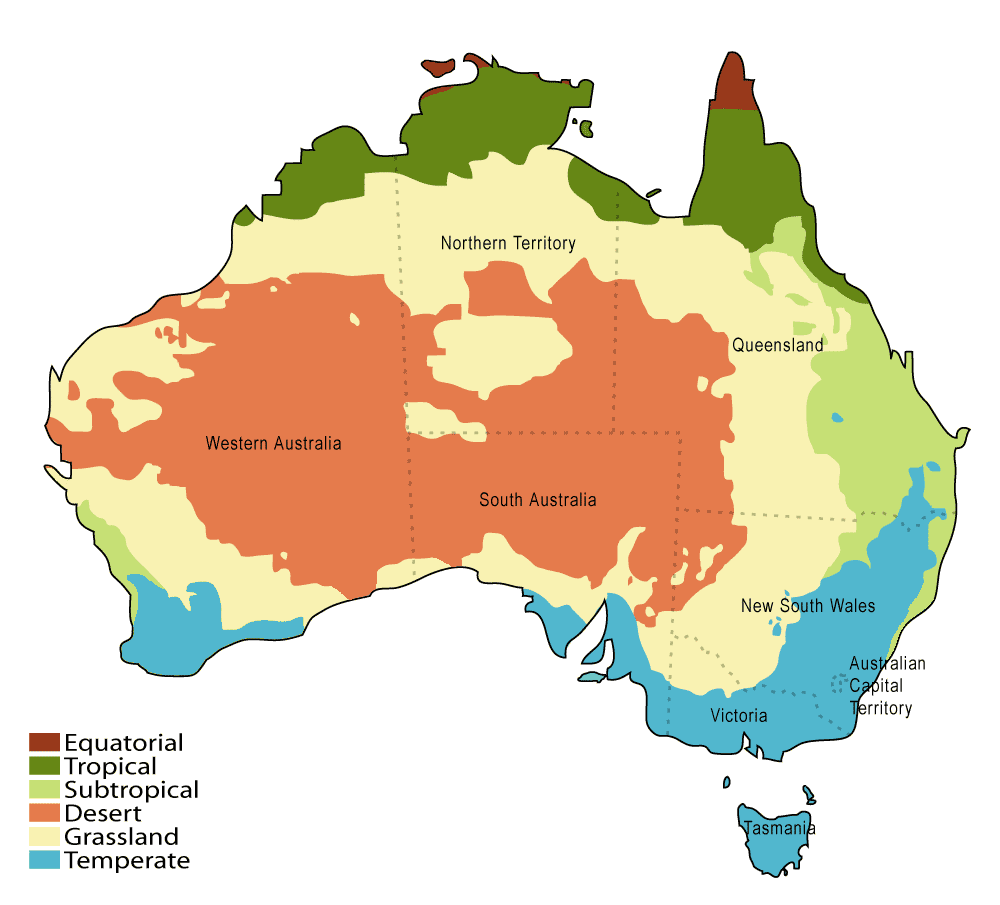


**Figure 1: Australian climate map**

*Note.* The Australian Bureau of Meteorology climate classification. Licences under the [Creative Commons](https://en.wikipedia.org/wiki/en:Creative_Commons) [Attribution-Share Alike 3.0 Unported](https://creativecommons.org/licenses/by-sa/3.0/deed.en) licences.

**Appendix 3**

**Quality and risk of bias assessment for all included studies**

| Study | Sample frame appropriate to address target population? | Appropriate sample of study participants? | Was the sample size adequate? | Were the study subjects and the setting described in detail? | Was the data analysis conducted with sufficient coverage of the identified sample? | Were valid methods used for the identification of the condition? | Was the condition measured in a standard, reliable way for all participants? | Was there appropriate statistical analysis? | Was the response rate adequate, and if not, was the low response rate managed appropriately? | Whether to include in Sys. Review/ meta-analysis | Comments/ reason for not including in the meta-analysis |
| --- | --- | --- | --- | --- | --- | --- | --- | --- | --- | --- | --- |
| (Morgan et al., 1996) | Yes (Single hospital-based study) | NA (Full cohort, not a sample) | NA | Study subjects with GAS septic arthritis are not described separately | Yes | Yes | Yes | Yes | Not mentioned | Systematic review only | Study on septic arthritis. |
| (Harris et al., 2011) | Yes (Single hospital-based study) | NA (Full cohort, not a sample) | NA | Yes | Yes | Yes | Yes | Time trends for GAS and risk factors not reported. | Not mentioned | Systematic review only | Study on beta-haemolytic streptococci bacteraemia |
| (Porter et al., 2013) | Yes (Single hospital-based study) | NA (Full cohort, not a sample) | NA | Study subjects were not described in detail. | Yes | Yes | Yes | Yes | Not mentioned | Systematic review only | Study on bacteraemia due to all causes |
| (Strachan et al., 2011) | Yes (13 major  tertiary paediatric hospitals) | NA (Full cohort, not a sample) | NA | Yes | Yes | Yes | Yes | No detailed analysis for GAS empyema | Not mentioned | Systematic review only | Study on paediatric empyema |
| (Coe et al., 2023) | Yes (Single hospital-based study) | NA (Full cohort, not a sample) | NA | Yes | Yes | Yes | Yes | No detailed analysis for GAS bacteraemia | Not mentioned | Systematic review only | Study on bacteraemia due to all causes |
| (Gear et al., 2015) | Yes (cases from whole of NT) | NA (Full cohort, not a sample) | NA | Yes | Yes | Yes | Yes | Yes | Not mentioned | Systematic review only | Study on GAS bacteraemia |
| (Carapetis et al., 1999) | Yes (Single hospital-based study) | NA (Full cohort, not a sample) | NA | Yes | Yes | Yes | Yes | Yes | Not mentioned | Systematic review only | Study on GAS bacteraemia |
| (Williamson et al., 2023) | Yes (Single hospital-based study) | NA (Full cohort, not a sample) | NA | Yes | Yes | Yes | Yes | Yes | Not mentioned | Systematic review only | Study on GAS bacteraemia |
| (McRae et al., 2020) | Yes (seven major  paediatric centres in Australia) | NA (Full cohort, not a sample) | NA | Yes | Yes | Yes | Yes | Yes | Not mentioned | Systematic review only | Cannot get the denominator population |
| (Sivagnanam et al., 2015) | Yes (One local health district) | NA (Full cohort, not a sample) | NA | Yes | Yes | Yes | Yes | Yes | Not mentioned | Systematic review and meta-analysis |  |
| (Thomson et al., 2022) | Yes (Whole state of Victoria) | NA (Full cohort, not a sample) | NA | Yes | Yes | Yes | Yes | Yes | Yes (approximately 60%  coverage of eligible isolates in the state in  2009, and 80% in 2017) | Systematic review and meta-analysis |  |
| (O'Grady et al., 2007) | Yes (Whole state of Victoria) | NA (Full cohort, not a sample) | NA | Yes | Yes | Yes | Yes | Yes | Yes (active and passive surveillance and additional measures to capture all cases) | Systematic review and meta-analysis |  |
| (Thel K. Hla et al., 2023) | Yes (National Notifiable  Disease Surveillance System) | NA (Full cohort, not a sample) | NA | Yes | Yes | Yes | Yes | Yes | Not mentioned | Systematic review and meta-analysis |  |
| (Attwood & Spelman, 2021) | Yes (Single hospital-based study) | NA (Full cohort, not a sample) | NA | Yes | Yes | Yes | Yes | Incidence rates were not mentioned | Yes (4 (8.5%) incomplete records) | Systematic review only | Study on GAS bacteraemia |
| (Boyd et al., 2016) | Yes (cases reported to NT Notifiable  Diseases System) | NA (Full cohort, not a sample) | NA | Yes | Yes | Yes | Yes | Yes | Not mentioned | Systematic review only | Study period overlapped in Birrell et al, 2022 study |
| (Wright et al., 2021) | Yes (cases from whole of WA) | NA (Full cohort, not a sample) | NA | Yes | Yes | Yes | Yes | Yes | Yes (used many data sources) | Systematic review and meta-analysis |  |
| (Carapetis et al., 1995) | Yes (Single hospital-based study) | NA (Full cohort, not a sample) | NA | Yes | Yes | Yes | Yes | Yes | Not mentioned | Systematic review only | Population for denominator was not available to calculate disease incidence. |
| (Whitehead et al., 2011) | Yes (cases from whole of QLD) | NA (Full cohort, not a sample) | NA | Yes | No^1^ | Yes | Yes | Yes | No^2^ | Systematic review and meta-analysis | Only data for 2007 to 2008 were included in meta-analysis. |
| (Norton et al., 2004) | Yes (cases from whole of North QLD) | NA (Full cohort, not a sample) | NA | Yes (the authors were contacted to get details about the setting) | Yes | Yes | Yes | Yes | Not mentioned | Systematic review and meta-analysis |  |
| (Birrell et al., 2022) | Yes (cases from whole of NT) | NA (Full cohort, not a sample) | NA | Yes | Yes | Yes | Yes | Yes | Not mentioned | Systematic review and meta-analysis |  |
| (Tam et al., 2023) | Yes (cases from whole of SA) | NA (Full cohort, not a sample) | NA | Yes | Yes | Yes | Yes | Yes | Not mentioned | Systematic review only | Study only on NF |
| (Dinsmore, 2021) | Yes (5 hospitals from all states and territories) | NA (Full cohort, not a sample) | NA | No (no details of study participant) | No (only the number of cases mentioned) | Yes | Yes | No (only the number of cases mentioned) | Not mentioned | Systematic review only | Population for denominator was not available to calculate disease incidence. |
| (McRae et al., 2020) | Yes (5 paediatric tertiary referral hospitals from all states and territories) | NA (Full cohort, not a sample) | NA | Yes (there are details of the study participants but not state and territory wise information) | Yes | Yes | Yes | Yes | Not mentioned | Systematic review only | Population for denominator was not available to calculate disease incidence. |
| (Er et al., 2015) | Yes (Single hospital-based study) | NA (Full cohort, not a sample) | NA | No (age distribution and indigenous status not mentioned) | Yes | Yes | Yes | Yes | Not mentioned | Systematic review only | Study on GAS bacteraemia |
| (Haggie et al., 2020) | Yes (Single hospital-based study) | NA (Full cohort, not a sample) | NA | No (no details of characteristics of GAS empyema cases) | Yes | Yes | Yes | Yes | Not mentioned | Systematic review only | Study on empyema |
| (Ching et al., 2019) | Yes (Single hospital-based study) | NA (Full cohort, not a sample) | NA | Yes | Yes | Yes | Yes | Yes | Not mentioned | Systematic review only | Population for denominator was not available to calculate disease incidence. |
| (J. Oliver et al., 2019) | Yes (cases from whole of Victoria) | NA (Full cohort, not a sample) | NA | Yes | Yes | Yes | Yes | Yes | Not mentioned | Systematic review only | Study period overlapped in Thomson et al, 2022 study |
| (Jane Oliver et al., 2019) | Yes (cases from whole of Victoria) | NA (Full cohort, not a sample) | NA | Yes | Yes | Yes | Yes | Yes | Not mentioned | Systematic review only | Study period overlapped in Thomson et al, 2022 study |
| (Smith et al., 2006) | Yes (study included whole of QLD) | NA (all isolates, not a sample) | NA | No patient information (number of isolates were considered, not the number of episodes) | Yes | Yes | Yes | Yes | Not mentioned | Systematic review only | The study looked at the antimicrobial susceptibility testing and *emm* sequencing. |
| (Davis et al., 2011) | Yes (Single hospital-based study) | NA (Full cohort, not a sample) | NA | No (characteristics of study participant not described under each organism) | Yes | Yes | Yes | Yes | Not mentioned | Systematic review only | A study on all causes for sepsis. |
| (Wilson & Varadhan, 2020) | Yes (two tertiary referral centres in Newcastle) | NA (Full cohort, not a sample) | NA | Yes | Yes | Yes | Yes | Yes | Not mentioned | Systematic review only | Study is on GAS community acquired pneumonia |
| (Hassell et al., 2004) | Yes (Single hospital-based study) | NA (Full cohort, not a sample) | NA | Yes | Yes | Yes | Yes | Yes | Not mentioned | Systematic review only | Study on GAS necrotising fasciitis |
| (Norton et al., 2006) | Yes (This is a case control study) | NA (this is a case control study) | Sample size calculation not mentioned | No | Yes | Yes | Yes | Yes | Not mentioned | Systematic review only | Case-control study on STSS |
| (Chen et al., 2016) | Yes (two tertiary paediatric referral centres in Victoria) | NA (Full cohort, not a sample) | NA | Yes | Yes | Yes | Yes | Yes | Not mentioned | Systematic review only | Study on TSS due to all causes |
| (Douglas et al., 2020) | Yes (Single hospital-based study) | NA (Full cohort, not a sample) | NA | Yes | Yes | Yes | Yes | Yes | Not mentioned | Systematic review only | Study on bacteraemia |
| (Nguyen et al., 2023) | Yes (Single hospital-based study) | NA (Full cohort, not a sample) | NA | Yes | Yes | Yes | Yes | Yes | Not mentioned | Systematic review only | Study on GAS bacteraemia |
| (Lithgow et al., 2014) | Yes (Single hospital-based study) | NA (Full cohort, not a sample) | NA | Yes | Yes | Yes | Yes | Yes | Not mentioned | Systematic review only | Not a population-based study |
| (Abo et al., 2023) | Yes (5 paediatric hospitals in Victoria, QLD, WA, NT | NA (Full cohort, not a sample) | NA | Yes | Yes | Yes | Yes | Yes | Not mentioned | Systematic review and meta-analysis |  |
| (Goldsmith et al., 2024) | Yes (Whole state of Victoria) | NA (Full cohort, not a sample) | NA | Yes | Yes | Yes | Yes | Yes | Not mentioned | Systematic review only | Study period overlapped in Thomson et al., 2022 study |
| (Nguyen et al.) | Yes (Single hospital-based study) | NA (Full cohort, not a sample) | NA | Yes | Yes | Yes | Yes | Yes | Not mentioned | Systematic review only | Study on GAS bacteraemia only |
| (Xie et al., 2025) | Yes, Five tertiary or quaternary health networks in Melbourne and Sydney. | NA (Full cohort, not a sample) | NA | Yes | Yes | Yes | Yes | Yes | Not mentioned | Systematic review and meta-analysis |  |

^1^Although the study period was from 2004 to 2009, data from 2004 to 2006 were not included in incidence calculation as the numbers were small (33).

^2^Out of 125 cases identified, 99 were included in the analysis due to lack of information (21% incomplete records)

**Appendix 4**

**Distribution of the commonest reported emm-types in Australia according to states and territories**

| ***emm*-type** | **Proportion of *emm-*type as a %** | | | | |
| --- | --- | --- | --- | --- | --- |
|  | **NSW** | **Victoria** | **NT** | **QLD** | **Northern QLD** |
| 1 | 9.7 (Sivagnanam et al., 2015) | 21.3-32.6 (Ching et al., 2019; O'Grady et al., 2007; Oliver et al., 2019) |  | 8.3-11.7(Smith et al., 2006; Whitehead et al., 2011) | 5.5 -28.8 (Er et al., 2015; Harris et al., 2011; Norton et al., 2004) |
| 89 |  | 9.6-16.7 (Attwood & Spelman, 2021; Oliver et al., 2019) |  | 7 (Whitehead et al., 2011) | 4.5-5.5 (Harris et al., 2011; Norton et al., 2004) |
| 12 | 8.5 (Sivagnanam et al., 2015) | 7-8.8 (O'Grady et al., 2007) |  | 6.4 -8.3 (Smith et al., 2006; Whitehead et al., 2011) | 4 (Harris et al., 2011) |
| 4 | 8 (Sivagnanam et al., 2015) | 4.4-4.7 (O'Grady et al., 2007) |  | 15.3 (Whitehead et al., 2011) |  |
| 92 |  |  |  |  | 6 (Harris et al., 2011) |
| 8 |  |  |  |  | 4 (Harris et al., 2011) |
| 101 | 8.3 (Sivagnanam et al., 2015) |  | 6.1 (Boyd et al., 2016) |  |  |
| 113 | 8 (Sivagnanam et al., 2015) |  | 12.2 (Boyd et al., 2016) |  |  |
| 11 | 8 (Sivagnanam et al., 2015) |  |  |  |  |
| 66 | 8.5 (Sivagnanam et al., 2015) |  |  |  |  |
| 28 |  | 5-15.7 (O'Grady et al., 2007) |  | 5.8(Smith et al., 2006) |  |
| 3.1 |  | 5-11.6 (O'Grady et al., 2007) |  |  |  |
| 232 |  | 11.9 (Attwood & Spelman, 2021) |  |  |  |
| 108.1 |  | 9.5 (Attwood & Spelman, 2021) |  |  |  |
| 197 |  |  | 12.2 (Boyd et al., 2016) |  |  |
| 81.1 |  |  | 11 (Boyd et al., 2016) |  |  |
| 81 |  |  | 6.1 (Boyd et al., 2016) |  |  |
| 49 |  |  |  | 7 (Whitehead et al., 2011) |  |
| 18 |  |  |  |  | 6.4 (Norton et al., 2004) |
| 80 |  |  |  |  | 6.4 (Norton et al., 2004) |
| 114 |  |  |  |  | 5.5 (Norton et al., 2004) |
| 22 |  |  |  | 6.1 (Smith et al., 2006) |  |
